# Keloid treatments: an evidence-based systematic review of recent advances

**DOI:** 10.1186/s13643-023-02192-7

**Published:** 2023-03-14

**Authors:** Laura A. Walsh, Ellen Wu, David Pontes, Kevin R. Kwan, Sneha Poondru, Corinne H. Miller, Roopal V. Kundu

**Affiliations:** 1 Northwestern University Feinberg School of Medicine, Chicago, IL USA; 2 Department of Dermatology, Northwestern University Feinberg School of Medicine, Chicago, IL 60611 USA

## Abstract

**Background:**

Keloids are pathologic scars that pose a significant functional and cosmetic burden. They are challenging to treat, despite the multitude of treatment modalities currently available.

**Objective:**

The aim of this study was to conduct an evidence-based review of all prospective data regarding keloid treatments published between 2010 and 2020.

**Methods:**

A systematic literature search of PubMed (National Library of Medicine), Embase (Elsevier), and Cochrane Library (Wiley) was performed in November of 2020. Search strategies with the keywords “keloid” and “treatment” were performed by a medical librarian. The search was limited to prospective studies that were peer-reviewed, reported on clinical outcomes of keloid therapies, and were published in the English language between January 1, 2010, and November 24, 2020.

**Results:**

A total of 3462 unique citations were identified, of which 108 studies met inclusion criteria. Current literature supports silicone gel or sheeting with corticosteroid injections as first-line therapy for keloids. Adjuvant intralesional 5-fluorouracil (5-FU), bleomycin, or verapamil can be considered, although mixed results have been reported with each. Laser therapy can be used in combination with intralesional corticosteroids or topical steroids with occlusion to improve drug penetration. Excision of keloids with immediate post-excision radiation therapy is an effective option for recalcitrant lesions. Finally, silicone sheeting and pressure therapy have evidence for reducing keloid recurrence.

**Conclusions:**

This review was limited by heterogeneity of subject characteristics and study outcome measures, small sample sizes, and inconsistent study designs. Larger and more robust controlled studies are necessary to further understand the variety of existing and emerging keloid treatments, including corticosteroids, cryotherapy, intralesional injections, lasers, photodynamic therapy, excision and radiation, pressure dressings, and others.

**Supplementary Information:**

The online version contains supplementary material available at 10.1186/s13643-023-02192-7.

## Introduction

Keloids are dermal proliferations of fibrous tissue that most often arise at sites of cutaneous injury and have significant impact on quality of life. Although keloids are seen in all populations, the highest prevalence is in people of color with an estimated incidence of 4–16% [1, 2]. These growths represent the most robust form of abnormal wound healing, presenting as raised, firm lesions that extend beyond the margins of original injury [2]. Several etiological factors have been proposed, including genetic and hormonal influences [3]. Increased wound tension has also been associated with keloid formation, although body locations with limited tension such as the earlobe are similarly affected [4].

Multiple hypotheses have been proposed for keloid formation. Though the pathogenesis of keloids is not fully understood, it likely involves the dysregulation of complex inflammatory pathways [5]. Proinflammatory cytokines IL-6 and -8 have been shown to increase scarring, while similarly, a decrease anti-inflammatory IL-10 increases scarring [6]. Keloidal fibroblasts and inflammatory cells may drive keloid formation by dysregulation of normal collagen turnover. Keloids are characterized by an increased ratio of type 1 to type 3 collagen deposition in a haphazard pattern with increased fibroblast proliferation rates and increased sensitivity to growth factors [6, 7]. Differences in growth factor production could be due to epithelial-mesenchymal interactions, retention of fetal proliferative pathways, or the hypoxic keloidal tissue environment. Tissue tension has also been implicated as mechanical tension is a driver of fibroblast activity and formation of collagen. Certain inherited human leukocyte antigen subtypes have been associated with keloids, suggesting an abnormal immune response to dermal injury as a cause of keloids. Lastly, dermal injury causing an immune response to sebum, leading to cytokine release stimulating mast cell infiltration and fibroblast activity, has been suggested given the predilection for keloids to form in sites of increased density of pilosebaceous units [7].

Keloids pose a significant functional and cosmetic burden. They are often pruritic or painful [8]. Additionally, they can introduce tension in adjacent tissue and cause restrictions in normal movement. The psychosocial effects of developing disfiguring scars have also been repeatedly demonstrated [9, 10]. Unfortunately, keloids do not regress spontaneously and are often refractory to treatment.

Current treatment options include intralesional and topical therapies, surgical interventions, radiation, and laser-based therapies [11–13]. Intralesional corticosteroids are a mainstay of treatment, although other injectables include bleomycin, 5-flourouracil, botulinum toxin type A, verapamil, avotermin, IL-10, mannose-6-phosphate, and insulin. Topical therapies include imiquimod and mitomycin C. Surgical excisions are often paired with a combination of these adjuvant pharmacotherapies, and there is ongoing innovation in keloid excision and wound closure technique. Radiation therapies include external-beam radiation and interstitial brachytherapy administered at low- or high-dose rates [13]. Pulsed dye laser (PDL), cryotherapy, and pressure dressings are often utilized, as well as over-the-counter silicone sheets and topical vitamin E creams. Despite the myriad of proposed treatment options, keloids continue to pose a therapeutic challenge, and an updated body of evidence-based recommendations to guide disease management is lacking.

## Objective

The objectives of this systematic review were to examine the evidence from the past decade for the treatment of keloids, determine the efficacy and limitations, and recommend areas for improvement.

## Methods

This systematic review of the relevant literature on keloid treatments was conducted according to methods outlined in the Cochrane Handbook and reported according to the recommendations from the Preferred Reporting Items for Systematic Reviews and Meta-Analyses (PRISMA) guidelines.

### Search strategy

A medical librarian (C. M.) created the search strategy to investigate therapies for keloid treatment published in English between the years 2010 and 2020. On November 24, 2020, searches were conducted on PubMed (National Library of Medicine), Embase (Elsevier), and Cochrane Library (Wiley) using keywords and subject headings related to “keloid” and “treatment.” The full search strategy is available at 10.18131/g3-b39v-s030.

### Inclusion criteria

Articles were included if they were peer-reviewed, had a prospective study design (including non-randomized interventional studies and randomized controlled trials), reported on clinical outcomes of keloid treatments, and were published in English between January 1, 2010, and the day searches were conducted (November 24, 2020).

### Screening and study selection

Studies from the search result were downloaded into an EndNote database. Two reviewers independently screened titles and abstracts of all obtained studies, ensuring studies met the inclusion criteria. Any disagreements were then consulted with a third independent reviewer. Full texts of studies that were included by title and abstract screening were further reviewed, again independently by the two reviewers. Any disagreements were also consulted with a third independent reviewer as needed.

### Risk-of-bias assessment

Risk of bias for studies that were classified as randomized controlled trials was evaluated with the RoB 2: a revised Cochrane risk-of-bias tool for randomized trials [14]. Five categories of bias — randomization process, deviations from intended interventions, missing outcome data, measurement of the outcomes, and the selection of reported outcomes — were assessed using the RoB 2 algorithm and classified as low risk, some concerns, high risk, or no information.

For studies that were non-randomized interventional trials, the risk of bias in non-randomized studies of interventions (ROBINS-I) assessment tool was used to evaluate the risk of bias in seven categories: confounding, selection of participants, classification of interventions, deviations from intended interventions, missing data, outcome measurement, and selective reporting [15]. The ROBINS-I guide was used to grade each category as low risk, moderate risk, serious risk, or no information.

Figures of the risk-of-bias results were created using the risk-of-bias VISualization (robvis) online tool [16].

### Data extraction

Two reviewers independently extracted data from the studies in the EndNote database. The following data were extracted as follows:

1. Publication details: Authors and date of publication
2. Study design: I.e., randomized control trial, single- or double-blind, split-scar study
3. Participants: Number of participants and demographics
4. Type of treatment or intervention
5. Outcomes including subject- and physician-reported responses to treatment, objective measures of treatment, recurrence rates, follow-up time, and adverse events.

### Data synthesis

We were not able to pool data from multiple studies given the heterogeneity of measurements used for quantifying outcomes. Data extracted from eligible studies were analyzed using a narrative approach. This synthesis aimed to provide an evidence-based review of all prospective data regarding keloid treatments and outcomes in the last decade.

## Results

### Overview

**Fig. 1:**
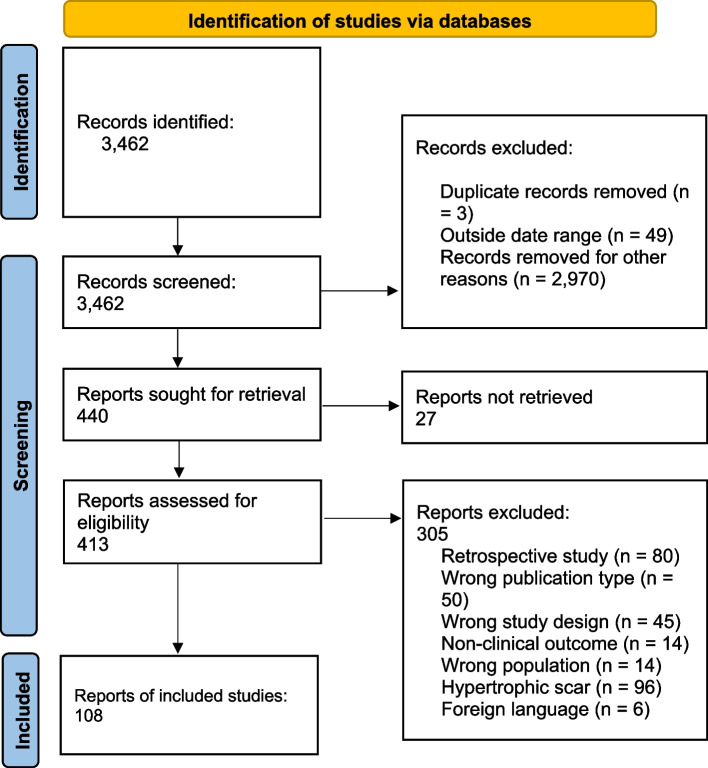
Flow diagram

There were 3462 articles included in the literature search. Screening of titles and abstracts yielded 440 articles for full-text evaluation, of which 108 were included, 305 were excluded, and 27 did not have full texts available to obtain (Fig. 1). Exclusion reasons included retrospective study design (80), wrong publication type (50), wrong study design (45), nonclinical outcome (14), wrong population (14), hypertrophic scar (96), and foreign language (6).

The total sample size was 4552 subjects (range of 6–240). The follow-up times varied from 4 weeks to 10 years. There were 37 randomized studies, 4 split scar studies, and 1 placebo-controlled studies.

**Fig. 2:**
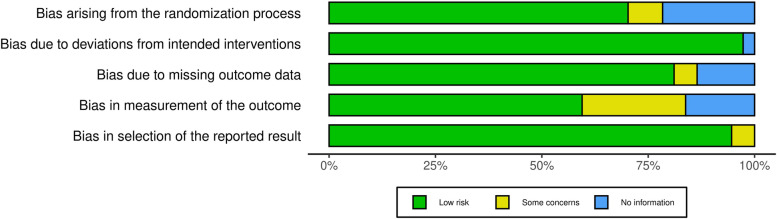
Risk-of-bias summary for randomized controlled trials assessed with RoB 2

**Fig. 3:**
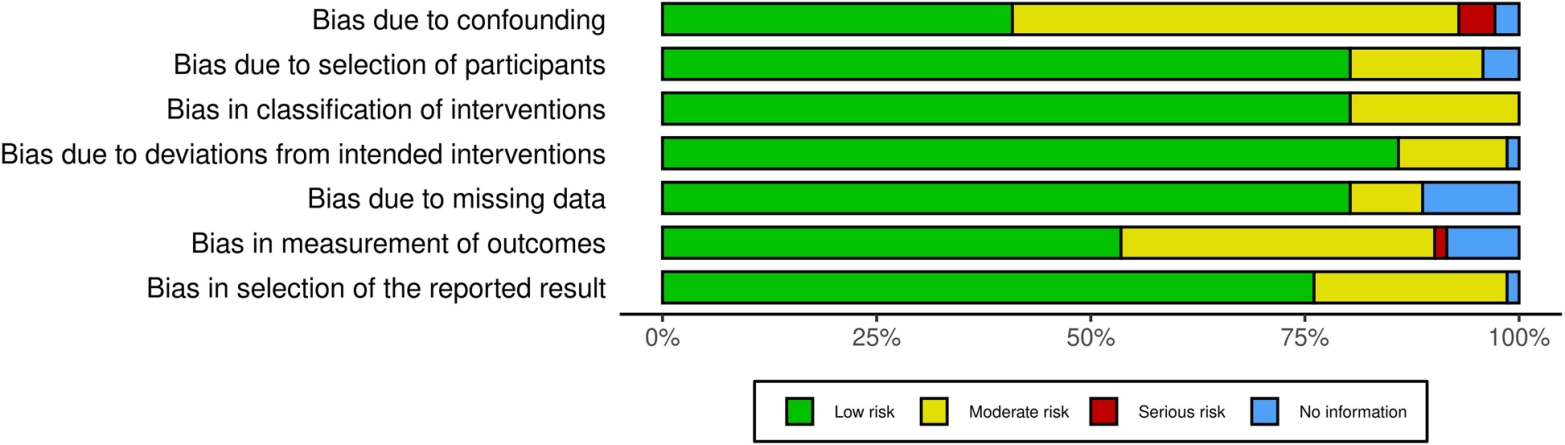
Risk-of-bias summary for non-randomized interventional studies assessed with ROBINS-I

Risk of bias in the 37 randomized controlled trials was low overall throughout the domains assessed in RoB 2 (Fig. 2). The measurement of outcomes domain had the highest proportion of studies with some concerns of bias, mainly due to lack of evaluator blinding and differences in timeframe of follow-up amongst the interventions (see Additional file 1 for the RoB 2 assessment for each study). Similarly, majority of non-randomized interventional studies were rated as low or moderate risk of bias with the ROBINS-I algorithm (Fig. 3). Only 4 out of the 71 non-randomized interventional studies had some components of serious risk of bias (see Additional file 2 for the ROBINS-I assessment for each study).

### Corticosteroids

Intralesional corticosteroids are the most commonly used nonsurgical treatment for keloids (Table 1). Intralesional triamcinolone acetonide (IL TAC) 10–40 mg/ml is most ubiquitous and induces keloid regression through a variety of proposed mechanisms including suppression of dermal inflammation, reduction of oxygen delivery to the wound bed via vasoconstriction, and antimitotic activity in keratinocytes and fibroblasts [17]. In review of 19 articles, there was unanimous clinical improvement in keloids with intralesional corticosteroid treatment. However, the degree of improvement and its relationship with treatment characteristics such as dosage, frequency, and timing of injections were variable [18–36].

**Table 1:** Topical and intralesional corticosteroids

First author, year	Study design	Treatment	Duration	*N*	Outcome(s)	Follow-up time mean (range)	Adverse events	Comments
Acosta, 2016 [18]	P	Intralesional (IL) triamcinolone acetonide (TAC)	Months 0 and 3 and monthly until optimal improvement (1–5 injections, median = 2)	25	Volume decreased an average 82.7% (*p* < 0.001) 4% recurrence	30 months (18–53)	Central depression (12.5%), telangiectasia (20.8%), fat deposits	In children 6–14 yo; ultrasound monitored volume
Aluko-Olokun, 2014 [19]	P	IL TAC	q2wks for maximum 6 months until flattening	52	Complete flattening in 88.46% of sessile vs 0% of pedunculated	18 months	Not stated	Injection with metal dental syringe
Aluko-Olokun, 2015 [20]	P	IL TAC dental syringe vs IL TAC hypodermic syringe	One injection	88	9.1% flattened vs 2.3% (*p* < 0.05)	25.2 mo (8–48)	Not stated	
Aluko-Olokun, 2016 [21]	P	IL TAC	Once	16	Reduction in volume greatest within the first 2-week post injection	6 weeks	Not stated	Investigated optimal frequency of administration
Aluko-Olokun, 2018 [22]	P	Post-excision IL TAC vs pre and post-excision IL TAC	q2wks for 5 months	18	No recurrence in either group	10 years	Not stated	Pedunculated earlobe keloids
Bashir, 2015 [23]	RCT	Intraop IL TAC + excision vs intraop and post-excision IL TAC	Once or at months 1 and 2 postop	70	No difference in recurrence rate	17.44 months (12–26)	Fewer complications w/single injection (8.5%) vs. postop IL TAC (23%): atrophy, telangiectasia, hypopigmentation, wound dehiscence	Helix keloids on female patients post-piercing
Brown, 2020 [24]	P	Full-thickness skin graft + IL TAC	Once	10	No recurrences reported	14 months	Not stated	Ear lobule keloids only
Cai, 2020 [36]	P	IL TAC	Q1wk for 4 weeks	51	Dermal thickness decreased by 39.0%	None	3 patients w/telangiectasia and 8 patients w/mild pruritus and pain	High-frequency ultrasound used for monitoring of keloids
Chua, 2019 [25]	SB, RCT	IL TAC to wound closure after excision	Once	150	N/a Trial protocol ongoing	12 months	Not stated	Pregnant women with keloid scars from a previous C-section
Dos Santos, 2015 [26]	SB, RCT	Excision vs preop IL TAC + excision	q1wk for 3-week preop	15	No significant difference in reduction of keloid dimensions	6 months	None	Earlobe keloids only
Farkhad, 2012 [27]	P	Group 1: IL TAC Group 2: IL TAC + silicone gel or sheet Group 3: excision + IL TAC	Unclear	44	Group 1 “good” result Group 2 “good result” Group 3 2 of 3 with recurrence	9–24 months	Not stated	No objective results, reported group 2 with best results
Huu, 2019 [28]	P	IL TAC 7.5 mg/cm^2^ vs 15 mg/cm^2^	Q4wks up to 6 times or clinical success	65	Lower dosage achieved “good” or “quite good” results in 90.7% of cases vs 68.7% of cases (*p* < 0.05)	None	3% ulceration, 5.6% menstrual disorder, 3.0% hypertension vs. 18.6% ulceration, 6.4% acne, 25% menstrual disorder, and 3.1% hypertension	Keloid respond graded as “good,” “quite good,” and “poor” based on criteria of Henderson and El-Tonsy
Kaushal, 2020 [29]	P	IL TAC vs IL radiofrequency (RF)+ IL TAC	Q3wks for 15 weeks	60	Equally efficacious and safe; RF and IL TAC fewer recurrences at 6 months	18 weeks	TAC: 13.3% pain, 16.6% hypopigmentation RF + TAC: 16.6% pain + ulceration, 13.4% pain, 10% atrophy, and 10% hypopigmentation	
Nor, 2017 [30]	RCT	Group 1: topical clobetasol propionate 0.05% cream under occlusion with silicone dressing Group 2: IL TAC 40 mg/ml	Group 1 daily for 3 months; G2 monthly for 3 months	17 patients (34 scars)	No significant difference in keloid improvement between group	None	Significantly more adverse effects: erythema (41.2 vs. 17.6%), hypopigmentation (35.3 vs. 23.5%), telangiectasia (41.2 vs.17.6%), skin atrophy (23.5 vs. 5.9%) in group 2 vs. group 1	
Schwaiger, 2017 [31]	P	Cryotherapy + IL TAC	Q1mo for 4 months	15	34.4% average decrease in volume, 41.3% average decrease in height	None	8 patients telangiectasia, 4 hyperpigmentation, and 1 ulceration	3D topographic imaging device and ultrasound monitored keloid volume and elevation
Tan, 2019 [32]	Intraindividual controlled, P	First phase: TAC embedded dissolving microneedle arrays (MNA) 0.025 mg Second phase: 0.1 mg	Daily for 30 days	Phase 1: 27 Phase 2: 17	Significant reduction volume, greater at higher dose, but increased to baseline 4 weeks after treatment	4 weeks	Not stated	Primary outcome was keloid volume as assessed by 3D scanner
Tey, 2017 [33]	P, SB, intraindividual controlled	TAC embedded dissolving hyaluronic acid MNA	4 weeks, self-applied	28	Transient decrease in volume that increased near baseline at 8 weeks, improved itch and pain with MNA	8 weeks	None	
Tey, 2018 [34]	P, SB, intraindividual controlled	TAC embedded (0.015 mg/patch then 0.1 mg/patch) dissolving hyaluronic acid MNA	4 weeks, self-applied	27 for low dose then 17 high dose	Transient decrease in volume (7.7% low dose, 12.9% high dose) at completion, increased near baseline at 8-week f/u, improved itch and pain with MNA	8 weeks	None	
Tripoli, 2015 [35]	P	Radical excision + IL TAC intraop and once postop vs radical excision	1-month postop	18	No recurrences with IL TAC, excision only with 6/9 recurrences	2 years	None	External ear keloids only

*P* prospective trial, *SB* single blind, *RCT* randomized controlled trial, *IL* intralesional, *TAC* triamcinolone acetonide, *yo* year old, *mo* month, *q2wks* every 2 weeks, *MNA* microneedle array, *RF* radiofrequency

In terms of dosing, 20–40 mg/ml of triamcinolone acetonide was most commonly investigated (8 of16 studies). Notably, a study by Huu et al. compared IL TAC 7.5 and 14 mg/cm^2^ and found a larger proportion of “good” and “quite good” results in the smaller dosage group; however, the size and characteristics of the studied keloids were not specified [28]. Frequency of treatments ranged from single injections to weekly and monthly injections. Aluko-Okun et al. (2016) studied optimal TAC dosing and observed the greatest reduction in keloid volume with 2-week treatment intervals [21].

Intralesional TAC was combined with surgical excisions in several studies with mixed results. Tripoli et al. reported no recurrences in subjects treated with two dosages of TAC after radial excision at their 2-year follow-up [35]. This is compared to the 9 controls who were excised without TAC and demonstrated a 67% recurrence rate. However, Dos Santos et al. compared excision +/− 3 weeks of preoperative 20-mg triamcinolone hexacetonide and found no significant difference in keloid dimensions at 6-month follow-up [26]. Bashir studied intraoperative TAC vs. intraoperative and postoperative TAC in 70 subjects and found no significant difference between the two groups [23]. Finally, when IL TAC 20 mg/ml was combined with intralesional radiofrequency in a cohort of 60 subjects, Kaushal et al. reported fewer recurrences at 6 months compared to IL TAC alone [29].

In addition to treatment parameters, keloid response is likely influenced by lesion characteristics. Aluko-Olokun et al. (2014) compared response of sessile vs. pedunculated lesions to TAC 10 mg and found a lack of response by pedunculated lesions compared to flattening of 23 of the 26 treated sessile lesions [19].

While topical steroids are less commonly used in the treatment of keloids, Nor et al. compared IL TAC 40 mg/ml monthly for 3 sessions to daily topical clobetasol propionate 0.05% cream under occlusion with silicone dressing [30]. There was no significant difference in reduction in keloid size; however, topical treatment resulted in significantly fewer adverse effects.

Finally, there is innovation in TAC drug delivery modalities, including a metal syringe and drug embedded microneedles. The metal syringe was proposed by Aluko-Olokun et al. as a new delivery system to address the issues of syringe failure and inadequate drug delivery to firm lesions [20]. Dissolving microneedles are self-administered once a month, empowering patients in their own care and reducing the inconvenience of frequent office visits. Initial studies suggest that these alternate delivery methods yield superior results compared to traditional plastic syringes. However, for TAC embedded microneedle arrays (MNAs), the volume decrease seems to be transient and not a durable response [33, 34].

### Cryotherapy

Cryotherapy or cryosurgery is a long-standing technique which relies on the reduction of temperature to cause irreversible cellular damage (Table 2). For treatment of keloids, studies have shown that cryotherapy transitions the keloidal fibroblasts towards a normal fibroblastic phenotype, increasing the ratio of type 3 to type 1 collagen in vitro [37, 38]. An additional advantage is that the decellularized matrix is left as a scaffold, possibly preventing recurrence. Cryosurgery alone has been shown to flatten keloids [39]. Intralesional verapamil, cryosurgery alone, or cryosurgery with intralesional TAC or verapamil all showed significant (*p* < 0.001) improvement in all VSS variables with no difference from cryosurgery with IL TAC [40]. Similarly, Fraccalvieri showed that cryosurgery alone or in combination with shave removal led to a majority of subjects (83% of 76 subjects) experiencing a 75–82% decrease in keloid height [41]. A smaller study of 12 subjects showed that a combination of shave removal, cryosurgery, and IL TAC had only 1 recurrence with 75% of subjects seeing a significant reduction in thickness [42]. Additionally, a combination of surgical excision, cryosurgery, and platelet-rich plasma (PRP) led to 70% of the 50 subjects observing improvement in keloid height and a recurrence 6 lesions after 7 months of follow-up [43].

**Table 2:** Cryotherapy

First author, year	Study design	Treatment	Duration	*N*	Outcome(s)	Follow-up time	Adverse events	Comments
Abdel-Meguid, 2015 [44]	P, RCT	Contact vs IL cryosurgery	Q3-4wks until flattening or max 6 sessions	35 vs 31	48.5% vs 83.9% complete flattening (*p* < 0.05)	3 months after final treatment	Pain, blistering, hypopigmentation	
Azzam, 2018 [43]	P	Excision + PRP + cryosurgery	Once	50	70% had height reduction (*p* < .05). 72% had improved scar pliability (*p* < .05) 6 of 37 (16.21%) recurred	12 months	Pain (44%), hypoesthesia (16%)	Auricular keloids only Vancouver Scar Scale (VSS)
Barara, 2012 [39]	P	Cryosurgery	q4weeks until 6 sessions or 75% flattening	30	Mean flattening 58.13% flattening after 6 sessions	6 months	Pain, dyspigmentation	
Bijlard, 2013 [45]	MC RCT	IL cryotherapy vs excision + IL TAC or excision + RT	Repeat at 3 months vs IL TAC at 2, 8, 12 weeks; vs 1 day RT	NA	NA	52 weeks	Not stated	Study protocol
Bijlard, 2018 [46]	MC RCT	IL cryotherapy vs excision + IL TAC; IL cryotherapy vs excision + RT	Repeat at 3 months vs IL TAC at 2, 8, 12 weeks or 1 day RT	26	No difference in primary keloids: IL cryo vs excision + IL TAC; excision + RT in resistant keloid improved appearance (POSAS) and symptoms, but IL cryotherapy did not	52 weeks		Terminated prematurely
Careta, 2013 [42]	P	Shave removal, cryosurgery, and IL TAC	IL TAC at 30 days	12 (13 keloids)	80% thickness reduction in 75% of patients, 1 recurrence	Mean 12 months	Not stated	Earlobe keloids
Fraccalvieri, 2016 [41]	P	Shave removal + cryosurgery vs only cryosurgery	once	153	94% complete smoothing (shave + cryo) vs 83% had 75–82% decrease in height (cryo)	12–72 months	Dyschromia, dystrophic scars	Groups were not compared
Jannati, 2015 [40]	RCT	Group 1: IL TAC+ cryotherapy Group 2: IL verapamil + cryotherapy Group 3: IL verapamil Group 4: cryotherapy	q3wks until flattening or 6 months	80	All groups with significant improvement in all VSS variables	1 year	Group 1: telangiectasia, atrophy, dyspigmentation, menses problems Group 4: dyspigmentation, bullae	
Mourad 2016 [47]	RCT	Spray versus IL cryotherapy	Q2wks for 10 sessions vs 6 sessions	50	Clinical improvement very good for 72% versus 96% (*p* = 0.02)	6 months after last session	Pain, blistering, infection, delayed wound healing, hypopigmentation	
Patni, 2017 [48]	P	IL cryotherapy	Repeat at 8–and 16weeks prn	15 (20 keloids)	POSAS with significant improvement; 50% with scar surface reduction of about 92%	1 year	Pain, erythema, bulla, hypopigmentation	
Van Leeuwen, 2014 [49]	P	Intralesional cryotherapy with argon gas		25 (30 keloids)	Volume reduction of 62%, POSAS improved 32%, 17% recurrence	1 year	Pain, blistering, wound dehiscence, hypopigmentation	

*P* prospective trial, *MC* multicenter, *SB* single-blind, *RCT* randomized controlled trial, *IL* intralesional, *TAC* triamcinolone acetonide, *PRP* platelet-rich plasma

Intralesional cryotherapy was first introduced in 1993 [50]. Patni et al. showed that with up to three sessions of intralesional cryotherapy, subjects saw a significant improvement of POSAS, and 50% of subjects saw a scar surface reduction of about 92% [48]. Additional recent investigation in the field of keloid treatment has compared intralesional cryotherapy to open spray cryotherapy. Mourad et al. and Abdel-Meguid et al. both showed that intralesional cryotherapy improved clinical appearance of keloids [44, 47]. However, a randomized trial by Bijlard et al. was terminated prematurely due to intralesional cryotherapy having inferior results to excision and IL TAC for primary keloids and excision and RT for resistant keloids [46]. A new innovation to intralesional cryotherapy is the use of argon in place of liquid nitrogen. The benefit is more controlled and accurate freezing and has a well-established history of use within the field of oncology. Van Leeuwen et al. showed a volume reduction of 62% [49]. However, further comparative studies will likely be required for such a technique to become more widely adopted.

### Intralesional injection

Many non-corticosteroid intralesional injections and combination treatments have been studied for keloid treatment including verapamil hydrochloride, 5-fluorouracil (5-FU), bleomycin, botulinum toxin A (BTA), hyaluronidase, and platelet-rich plasma (PRP) (Table 3). In many cases, TAC was used as the control group treatment when investigating these other agents.

**Table 3:** Intralesional Injection (with or without intralesional corticosteroids)

First author, year	Study design	Treatment	Duration	*N*	Outcome(s)	Follow-up time	Adverse events	Comments
Abou-Taleb, 2020 [51]	P	IL verapamil (2.5 mg/ml)	Q3wks until complete flattening or 6 sessions	43	Significant decrease in mean VSS score (*p* < 0.001) Recurrence in 20.9% of cases	Up to 3 months	Post-procedure pain in 83.7% Post-procedure pruritus in 9.3%	
Aggarwal, 2018 [52]	RCT	Group 1: IL TAC (40 mg/ml) Group 2: IL hyaluronidase (HA) 1500 IU/ml + TAC 40 mg/ml (1:1) Group 3: IL verapamil (2.5 mg/ml) Group 4: RF Group 5: RF + IL TAC (40 mg/ml)	Groups 1, 3, 5: q3wks for 8 sessions or complete flattening Group 4: q6wks for 4 sessions or complete flattening	80	Complete clearance: 75% Group 1: 68.75% Group 2: 0% Group 3: 11.76% Group 4, 75% Group 5 (*p* < 0.001)	5 months	Groups 1, 2, and 5: atrophy and pigmentary (least in group 2 (*p* value < 0.001)); telangiectasia (group 1), urticaria (groups 3); ulceration + secondary infection in groups 4 and 5 (35.29% and 25%) (*p* value *<* 0.001)	Clearance: height reduced to 1 mm or less
Ali, 2020 [53]	RCT	Group A: IL 5-FU (50 mg/ml) Group B: IL 5-FU + IL TAC (40 mg/ml) (9:1)	q1wk for 4weeks, then twice a month for 2 months then q1mo until flat or max of 3 months	60	Efficacy higher in group B (86.7% vs. 60% *p* = 0.020)	6 months	Skin necrosis in an unspecified number of cases	Effectiveness: more than or equal to 50% reduction in initial height Group B effectiveness was higher only in ≤ 40 years (*p* = 0.013)
Danielsen, 2016 [54]	DB, RCT, split scar controlled	Group 1: excision + IL TAC (10 then 5 mg/ml) Group 2: excision + IL verapamil (2.5 mg/ml)	Q1mo for 4 months	14	Higher recurrence verapamil-treated half 6/14 (*p* = 0.01)	12 months	4 subjects w/atrophy IL-TAC half	The study was terminated early due to superior results in group 1
El Kamel, 2016 [55]	P	Keloidectomy with core fillet flap + IL verapamil (2.5 mg/ml)	q2wks for 3 months	16	71.4% with no recurrence 14.3% recurrence at wound bed	18 months	Partial flat tip necrosis in 10.5% 14.3% hypertrophic scar at incision	Earlobe keloids only
Gamil, 2020 [56]	RCT, intraindividual controlled	Group 1: IL BTA (2.5 IU/cm^3^) one side of the body and IL TAC to other side Group 2: IL BTA + IL TAC	q1mo for 3 sessions	50	Group 2 shows significantly greater reduction in keloid surface area vs IL TAC	6 months	Group 1: 11.5% pain w/injection, 7.7% skin atrophy Group 2: none	Scar Evaluation Scale (SBSES) and color Doppler ultrasound (CDS) used to evaluate keloids
Hewedy, 2020 [57]	RCT	Group A: IL TAC (20 mg/ml) + PRP 1 week afterwards Group B: IL TAC (20 mg/ml)	IL TAC q3wks for 4 sessions	40	Statistically significant better improvement in VSS in group A than in group B after treatment (*p* = 0.026)	3 months	Significantly higher atrophy and hypopigmentation in subjects of group B vs A (*p* = 0.01 and .014)	
Huu, 2019 [28]	P	IL bleomycin (15 units)	Q4wks for an average of 4 times	120	14% recurrence at follow-up	18 months	Hyperpigmentation in 56.7%, blistering in 78.3%, ulceration in 5.8%	VSS used to quantify treatment response
Ismail, 2020 [58]	RCT	Group A: IL BTA (2.5 U/cm^3^) Group B: IL 5-FU (50 mg/ml)	Q1mo until flattening or 6 sessions	69	Greater flattening group A vs group B (*p* = 0.04) 8.8% vs 31.4% recurrence group A vs B (*p* < 0.05)	Up to 3 months	Group A: hypopigmentation in 5.9% Group B: hyperpigmentation in 14.3% and hypopigmentation in 2.9%	6 patients had multiple keloids and received different treatments in different lesions
Khan, 2019 [59]	RCT	Group A: IL bleomycin (1.5 IU/ml) Group B: IL TAC (40 mg/ml)	q4wks for 6 months	164	Decrease in POSAS score was significantly larger in group A Efficacy 82% vs 70% group A vs B (*p* = .0069)	None	Group A: hyperpigmentation in 70%, and ulceration in 27% Group B: atrophy in 70%, hypopigmentation in 29%, and telangiectasias in 21%	POSAS score used to quantify treatment response Efficacy: greater than 50% reduction in the POSAS score from baseline
Khare, 2012 [60]	P	Excision with 5-FU to excision margin and the wound bed vs. IL TAC	IL TAC q2wks	60	Recurrence rate with excision and 5-FU was 3.57% vs IL TAC 21.9%	1 year	Excision and 5-FU group with superficial necrosis in 11%, dehiscence in 7%, and local infection in 4%	Earlobe keloids
Khattab, 2020 [61]	P	IL verapamil (2.5 mg/ml) vs PDL then IL verapamil (2.5 mg/ml)	Q3 weeks for up to 8 sessions or flattening	40 (56 keloids)	PDL + verapamil showed a statistically significant greater reduction in height (*p* = 0.003) and pliability (*p* = 0.025)	24 weeks	Increase size, pain, purpura, hyperpigmentation, and depigmentation AE more frequent in PDL + verapamil (25%) compared to IL verapamil (5.36%)	VSS
Pruksapong, 2017 [62]	RCT	Control group: IL TAC (10 mg/m)l 7 days after suture removal Toxin group: BTA (1.5 μ/cm) 7 days after suture removal	Control group only: additional injections at 1, 3, and 6 months	25 (50 keloids)	VSS score in control group significantly lower than the toxin group at 6th month follow-up (5.33 ± 1.87 vs. 4.11 ± 1.96, *p* = 0.010)	6 months	Not stated	
Rasaii, 2019 [63]	DB, RCT, intraindividual controlled	Group A: IL TAC (20 mg/ml) + placebo Group B: IL TAC + IL BTA (20 U/ml)	q4wks for 3 sessions	40	No significant difference in therapeutic efficacy between groups	1 month	Not stated	VAS used to quantify treatment response
Reinholz, 2020 [64]	P	IL 5-FU (50 mg/ml) + IL TAC (40 mg/ml) (3:1)	q4wks for 4 treatments	50	All parameters in the patient score revealed significant improvement after treatment Keloid height and volume were reduced by 59.3% and 53.1% DLQI score showed improvement in QOL	12 months	Hyperpigmentation (36%), telangiectasia (24%), ulceration (20%), hyperpigmentation (12%)	Inclusion criteria included resistant to treatment after 3× cryotherapy + TAC Treatment response monitored by digital photography, three-dimensional phase shift rapid in vivo measurement of skin (PRIMOS) software, ultrasound and standardized questionnaires (POSAS, DLQI)
Sadeghinia, 2012 [65]	DB, RCT	Group A: IL TAC (40 mg/ml) Group B: 5-FU solution (50 mg/ml) dripped after 40 punctures per 5 mm^2^ (tattoo method)	q4wks for 12 weeks	40	Patient self-assessment, induration, pruritus were significantly better (*p* < .05) in Group B Better results were found for group B group (*p* < .05) per observer assessment	44 weeks	None	
Sagheer, 2016 [66]	RCT	Group A: IL 5-FU (50 mg/ml) Group B: IL TAC (40 mg/ml)+5-FU (1:9)	Monthly for 6 months	60	Group A with efficacy in 10 (33.3%) cases vs. 22 (73.3%) group B (*p* = 0.002.)	6 months	Not stated	Efficacy: 51–100% improvement (flattening and decrease in size of lesion
Saha, 2012 [67]	RCT	Group F: IL 5-FU (50 mg/ml) Group T: IL TAC (40 mg/ml)	Frequency unspecified, until satisfactory result	44	Both modalities of treatment were equally effective	Up to 1 year	Group F: ulceration, hyperpigmentation	
Saki, 2019 [68]	RCT, intraindividual controlleld	IL TAC (20 mg/ml) + cryotherapy Vs IL verapamil (2.5 mg/ml)+ cryotherapy	q3wks until flattening or 8 sessions	30	Statistically better improvement in height and pliability in the triamcinolone-receiving group compared with the verapamil-receiving group (P < 0.001).	24 weeks	TAC: hyperpigmentation and hypopigmentation Verapamil: hyperpigmentation	Scar evaluation at each stage was done by serial photographic records as well as by Vancouver Scar Scale
Saleem, 2017 [69]	RCT	Group A: IL 5-FU (50 mg/ml) + TAC (40 mg/ml) Group B: IL TAC (40 mg/ml)	q4wks until flattened or period of 12 weeks	100	Mean reduction in VSS was −71.18 (±8.69) in group A as compared to −50.80 (±8.59) in group B (*p* = 0.001)	12 weeks	No serious adverse effects	
Shaarawy, 2015 [70]	DB, RCT	Group A: IL TAC (10 mg/ml) Group B: IL BTA (5 IU/cm^3^)	Group A: q4wks for six sessions or complete improvement Group B: q8wks for 3 sessions or complete improvement	24	Significant decrease in the volume (*p* < 0.01), softening (*p* < 0.01) and decrease in height (*p* < 0.01), no significant difference between groups	None	Group A: skin atrophy and telangiectasia 25%	
Srivastava, 2017 [71]	SB, RCT	Group A: IL TAC (40 mg/ml) Group B: IL 5-FU (50 mg/ml) Group C: IL TAC (40 mg/ml) + IL 5-FU (50 mg/ml) (1:9)	q3wks for 24 weeks or resolution	60	There was a reduction in VSS all three groups	none	Telangiectasias and skin atrophy most frequently in group A. Skin ulceration was a common problem in group B	Resolved: when a total score of 2 or less was achieved on Vancouver Scar Scale (VSS)
Velurethu, 2017 [72]	P	IL 5-FU (50 mg/ml) + IL TAC (40 mg/ml) + IL HA (1500 units)	q4wks until complete flattening or a maximum six sessions	50 (60 keloids)	Significant improvement of POSAS at 12 weeks for all patients 65% with complete flattening after 4 sessions 2 recurrences at 6 months	6 months–1 year	Skin ulceration (13%), hypopigmentation (23%)	
Wilson, 2013 [73]	P	Excision w/ POD 9 IL 5-FU (50 mg/ml) and IL BTA (50 IU/ml)	Once	80	Recurrence rate of 3.75%	17–24 months	Pruritus (10%), pain (8.75%), burning (5%), hyperpigmentation (2.5%), dehiscence (1.25%), late scar widening (13.75%)	

*P* prospective trial, *SB* single blind, *DB* double blind, *RCT* randomized controlled trial, *IL* intralesional, *TAC* triamcinolone acetonide, *yo* year old, *mo* month, *qnwks* every n weeks, *HA* hyaluronidase, *IU* international unit, *5-FU* = 5-fluorouracil, *BTA* botulinum toxin A, *PRP* platelet-rich plasma, *PDL* pulsed-dye laser

Verapamil is a calcium channel blocker that suppresses extracellular matrix molecules formation and promotes collagen breakdown. It is commonly used in the concentration of 2.5 mg/ml when treating keloids. In several noncontrolled studies, verapamil treatment alone or in combination with keloidectomy or pulse dye laser (PDL) resulted in decreased VSS scores and positive clinical response [51, 55, 61]. However, intralesional verapamil was inferior when compared to IL TAC. In a double-blinded controlled trial comparing 4 monthly doses of verapamil to identically scheduled TAC 5 mg/ml in 14 keloid lesions, there was significantly higher recurrence rates at 12-month follow-up with a hazard ratio for recurrence of 8.44 ( 95% *CI* 1.62–44.05) [54]. In their intraindividual study, Saki et al. compared verapamil + cryotherapy to TAC 20 mg/ml + cryotherapy in opposite ends of the same lesion (a split scar study); results showed statistically greater reduction in height and improved pliability in the TAC group [68].

Bleomycin is an antineoplastic agent that causes necrosis of fibroblasts. Two studies investigated bleomycin and demonstrated its utility in keloid treatment [59, 74]. Khan et al. most robustly showed this effect in 164 keloids: 6 doses of monthly 1.5 IU/m was more effective than identically scheduled TAC 40 mg/ml, achieving 50% reduction in the POSAS score from baseline. This difference was independent of age, gender, Fitzpatrick skin type, the duration of keloids, or baseline POSAS score [59].

The antimetabolite 5-FU inhibits fibroblast proliferation through disruption of DNA replication. 5-FU is used independently and in combination with other treatments, most commonly IL TAC. Saha et al. compared 5-FU with TAC in 44 subjects and showed both were equally effective [67]. Ali et al., in a randomized controlled trial comparing 50 mg/ml 5-FU alone with combination 5-FU 50 mg/ml (0.9 ml) + 40 mg/ml TAC (0.1 ml), showed that reduction of mean keloid height after treatment was significantly greater in the combination group (*p* = 0.0008) [53]. Saleem et al. similarly showed a combination of TAC+5-FU had significantly greater improvement in VSS than TAC alone in 100 subjects [69]. Sagheer et al. demonstrated similar superiority of combination TAC 40 mg/dl (0.1 ml) and 5-FU 50 mg/ml compared to 5-FU alone [66]. Notably, adverse effects were not reported in either study; however, in another noncontrolled study, Reinholz et al. demonstrated local adverse effects in > 90% of their subjects, including hyperpigmentation, telangiectasia, and ulceration [64]. Srivastava et al. compared TAC vs. 5-FU vs. TAC + 5-FU and showed all improved VSS scores compared to baseline in 60 subjects [75]. Finally, Sadeghinia et al. compared intralesional TAC 40 mg/ml to 5-FU applied by a unique tattoo method [65]. In the latter group, 5-FU 50 mg/ml solution was dripped on each 1 cm^2^ of the lesions. Subsequently, 40 punctures per 5 mm^2^ were made followed by a second round of 5-FU drip application. This methodology theoretically allows for deeper and more even penetration of the drug and resulted in significantly decreased induration and pruritus and improved observer assessment by a blinded dermatologist with respect to overall improvement on a 5-point scale.

Botulinum toxin A (BTA) is a neurotoxin known for its paralytic effects. Its utility in keloid treatment may be related to reduction of muscular tension at wound sites and direct fibroblast regulation. No significant difference was found in 2 double-blinded controlled trials comparing 5 IU/cm^3^ to TAC 10 mg/ml and BTA 20 μ/ml to TAC 20mg/ml, respectively [63, 70]. Interestingly, in a head-to-head comparison between 5-FU 50 mg/ml and BTA 2.5 U/cm^3^, Ismail et al. showed significantly greater flattening by BTA (*p* = 0.04) [58]. As a combination therapy, Gamil et al. showed significantly (*p* = 0.0001) reduced keloid surface area in 24 keloids treated with intralesional BTA and TAC compared to 26 subjects treated with TAC or BTA alone [56].

The enzyme hyaluronidase catalyzes the breakdown of the mucopolysaccharide hyaluronic acid. Although it has been studied in the treatment of keloids, its mechanism of action is not clearly understood. Aggarwal et al. showed that TAC + 1500 IU/ml hyaluronidase had similar clinical efficacy compared to triamcinolone alone but fewer side effects (18.75% subjects developed atrophy with combination in comparison with 31.25% subjects with triamcinolone alone, *p* < 0.001, chi-square test) [52]. The author highlights that in the combination group, the TAC dosage was effectively halved, suggesting a synergistic effect of TAC and hyaluronidase combination treatment. Velurethu et al. showed a combination of intralesional 5-FU, TAC, and hyaluronidase every 4 weeks for 50 subjects with 60 keloids led to flattening in 65% and > 90% reduction in scar volume in 35% of keloids after 4 sessions [72]. Only two recurrences were observed at follow-up after 6 months.

PRP is autologous platelet concentrate that is used in a variety of conditions to promote wound healing, decrease pain, and combat inflammation. In an RCT comparing gold standard IL TAC 20 mg/ml every 3 weeks for 4 sessions to identically scheduled IL TAC followed by 1 injection of PRP, the latter was shown to have superior keloid response and fewer adverse effects [57].

In combination with keloid excision, intralesional treatment with the previous therapeutics is used to decrease recurrence rates. Khare et al. treated the wound bed and margin with 5-FU after excision for 28 subjects [60]. They observed a recurrence rate of 3.57% in the 28 treated subjects compared with a 21.9% recurrence rate over 1 year in the 32 control subjects treated with IL TAC. Similarly, Wilson et al. treated 80 subjects with excision followed by IL 5-FU and BTA 9 days post surgery and observed a recurrence rate of 3.75% [73]. Pruksapong et al. randomized 25 subjects with 50 keloids to keloid excision and then IL TAC or IL BTA [62]. Subjects receiving IL BTA had significantly (*p* < 0.010) decreased VSS.

### Light-based therapy

#### Lasers

Both ablative and non-ablative lasers have been proposed for the treatment of keloids (Table 4). Ablative lasers include the erbium (Er:YAG) laser and CO2 laser, and they cause local tissue destruction by targeting the water chromophore. Non-ablative lasers such as ND:YAG, diode lasers, and pulsed dye lasers (PDL) target melanin and/or hemoglobin. The mechanism by which lasers treat keloids is less clear and may include local damage to lesional blood vessels or direct fibroblast suppression. While lasers can be used as independent therapy for keloids, they are also being investigated in combination with therapeutics to assist in drug delivery and penetration. In our cohort of prospective studies, CO_2_ lasers were the most frequently investigated, followed by erbium ablative lasers, ND:YAG, diode lasers, and finally PDL.

**Table 4:** Laser therapy

First author, year	Study design	Treatment	Duration	*N*	Outcome (s)	Follow-up time	Adverse events	Comments
Abd El-Deyem, 2020 [76]	P, split side controlled	IL TAC (10 mg/ ml) versus 2940 nm Er:YAG laser with betamethasone and film covering immediately after	Q4 weeks for 4 treatments	30	VSS from 6.90 to 2.63 versus 2.07 (*p* > 0.05)	12 weeks after last session	Telangiectasia, atrophy, leukoderma, hyperpigmentation versus just hyperpigmentation	
Annabathula, 2017 [77]	P	Sequential fractional CO_2_, PDL, then Nd:YAG	Q4 weeks for 5 sessions	15	Improvement: 9% excellent, 9% good, 37% moderate, 27% with no change	6 months	none	4 patients lost to follow-up, 1 patient with increased size of keloid
Behera, 2016 [78]	RCT	CO_2_ then IL TAC (40 mg/ml) versus contact cryotherapy then IL TAC (40 mg/ml)	Q4 weeks for 3 months	60 (101 keloids)	38.89% versus 40.47% complete flattening (*p* = 1.00); 16.66% recurrence in CO_2_ treated	12 months	Infection, erythema, hypopigmentation (more with cryotherapy), erosion, pain, atrophy, telangiectasia, comedone; early side effects more common with CO_2_	
Chen, 2017 [79]	P	IL CS (diprospan) versus IL 5 FU and CS versus IL 5-FU and CS then 1,064-nm Nd:YAG	Q4 weeks for 3 sessions	62 (69 keloids)	Patient: excellent response 20% vs 58% vs 78% Blinded observer: excellent response 12% vs 48% vs 68%	3 months	Pain for all groups 36% atrophy and telangiectasia vs none vs initial purpura	
Garg, 2011 [80]	P	CO_2_ laser then IL TAC of 40 ng/ml	IL TAC Q3–4 weeks for 6 months	28 (35 keloids)	Regular follow-up: 11.7% recurrence Irregular follow-up: 75% recurrence	1 year, 6 months after final IL TAC	Erythema, infection, telangiectasia, atrophy, dyschromia,	5 patients lost to follow-up
Kassab, 2012 [81]	P	980-nm diode then IL TAC (40 mg/ml)	Q3 weeks for 2 to 5 sessions	12 (16 keloids)	12 out of 16 had > 75% reduction in size	12 months	Infection, hyperpigmentation	Earlobe keloids
Park, 2017 [82]	P, split side controlled	Er:YAG laser then IL TAC (10 mg/cm^3^) vs topical desoximetasone 0.25% ointment with 3 h occlusion using transparent film dressing	Q6 weeks for four sessions	10	Improvement in VSS, but no difference between sides	12 weeks after last treatment	Higher pain for IL TAC, telangiectasia	
Srivastava, 2019 [71]	RCT	IL TAC (40 mg/ml) vs IL verapamil (2.5 mg/ml) vs fractional CO_2_	Q3 weeks for 24 weeks or flattening	60	All groups improved VSS; IL TAC had fastest improvement	6 months	Pain, telangiectasia, atrophy vs none vs pain and charring	
Wang, 2020 [83]	P	Fractional CO_2_ laser then applied triamcinolone acetonide (40 mg/ml) with 4 h occlusion with transparent film dressing	Q4 weeks for 8 sessions	41	POSAS observer score 37.73 to 25.29 after treatment; patient 39.59 to 22.34 10.5% recurrence	24 months	Telangiectasia, hyperpigmentation	3 subjects lost to follow-up

*P* prospective trial, *RCT* randomized controlled trial, *IL* intralesional, *TAC* triamcinolone acetonide, *qn* weeks every *n* weeks, *PDL* pulsed-dye laser, *CS* corticosteroid

In their RCT of 60 subjects, Behera et al. found no significant difference in therapeutic response by keloids treated with 5 sessions of CO_2_ laser compared to cryotherapy, both in conjunction with IL TAC 40 mg/ml [78]. However, CO_2_ laser therapy yielded more frequent early adverse effects. A prospective study of 41 keloids treated with CO_2_ followed by topical TAC 40 mg/ml Q4 weeks for 8 sessions showed a recurrence rate of 10.5% at 24 months [83]. Garg et al. similarly showed a recurrence rate of 11.7% in subjects treated with CO_2_ with regular follow-up of IL TAC in 35 treated keloids [80]. Unfortunately, there were no studies of CO_2_ laser + IL TAC compared to IL TAC alone, precluding the direct evaluation of CO_2_ laser treatment. Srivastava et al. compared CO_2_ ablative laser alone compared to IL TAC 40 mg/ml alone and found no significant differences between keloid response but faster improvement in the IL TAC group [71].

In a split-side controlled study, Abd El-Deyem et al. demonstrated the superiority of fractional ablative 2940 nm Er:YAG laser-assisted delivery of betamethasone vs IL TAC 10 mg/ml alone [76]. The difference in steroid used between groups is a significant confounding variable. Conflicting results were reported in another study where no difference in clinical improvement was appreciated between keloids treated with Er:YAG laser and IL TAC 10 mg/ml versus topical desoximetasone 0.25% ointment with 3-h occlusion [84].

A prospective study of 62 subjects showed that the addition of 1064-nm Nd:YAG to IL disprospan and IL 5-FU resulted in superior results compared to either drug alone or the two combined (78% excellent responses vs. 58% and 20%) [79]. These results make a compelling case for Nd:YAG-assisted drug delivery. Annabathula et al. combined Nd:YAG, CO_2_, and PDL Q4 weeks for 5 sessions. In their 11 subjects whom completed the study, 5 showed minimal to no improvement, 4 moderate (26–50%), improvement, and 2 > 50% improvement based on size, color, and aesthetic impression by three blinded dermatologists [77].

Kassab et al. followed clinical improvement of earlobe keloids treated with 980 nm diode followed by IL TAC 40 mg/mL Q3 weeks for a variable 2–5 sessions [81]. While 7% of lesions shrunk at least 75% in size, the sample size was small (*n* = 16).

#### Photodynamic therapy

There is sparse but emerging evidence on the utilization of photodynamic therapy (PDT) in treating keloids and hypertrophic scars (Table 5). PDT is typically administered following the application of a photosensitizing agent such as 5-aminolaevulinic acid (ALA). While the mechanisms underlying the response of keloids to PDT are still under investigation, PDT is emerging as a potential adjunct therapeutic option for keloid treatment.

**Table 5:** Photodynamic therapy

First author, year	Study design	Treatment	Duration	*N*	Outcome(s)	Follow-up time	Adverse events	Comments
Bu et al, 2020 [85]	P (split scar control)	Excision + RT (5 Gy every 5 days) then split scar PDT	PDT Q1 wk for 4 treatments	10	VSS 7.20 vs 6.25 at 20 months (w/o vs w/PDT)	20 months	Pain, hyperpigmentation, blister	
Basdew et al., 2013 [86]	P, controlled	Excision + RT (9 Gy ×2) vs excision + PDT	Q6hrs vs interstitial PDT at 4 h, 6 h, and 3 days, later subjects received 6 q1wk topical PDT	34 subjects treated for 45 keloids	Observers POSAS 19.1 vs 24.6 (RT vs PDT) Independent observers POSAS 14.6 vs 18.6 (RT vs PDT)	64 vs 34.4 weeks (RT vs PDT)	Burning with interstitial PDT requiring IV opioids Topical PDT sessions required oral NSAIDs, morphine, or transdermal fentanyl	

*P* prospective trial, *RT* radiotherapy, *Gy* gray, *PDT* photodynamic therapy, *qn* every n

Basdew et al. conducted one of the first large-scale studies investigating the clinical use of PDT for keloid treatment, comparing surgical excision with either adjunctive interstitial brachytherapy or ALA applied to the wound bed followed by postoperative interstitial PDT using an inserted transparent catheter with a cylindrical diode laser diffuser [86]. Subjects and observers were more satisfied with results after brachytherapy than PDT; however, subjects had a positive general impression after PDT. Adverse effects of burning were present for all subjects during interstitial illumination treatments necessitating intravenous opioids. Topical PDT sessions were better tolerated. Bu et al. preformed a prospective trial comparing surgery and superficial X-ray radiation therapy vs. surgery, superficial X-ray radiation therapy, and PDT in the split scar study in 10 subjects [85]. Both treatments noted significant symptom reduction. Only 1 keloid was painful at baseline which was relieved in both treatment groups by 6-month follow-up but reappeared in the treatment of postoperative radiation alone at 20-month follow-up. One of the ten subjects experienced keloid recurrence at 20 months on both sides of the scar. Adverse effects of mild pain were noted with PDT as well as one blister developing after PDT. Mild hyperpigmentation was observed in 6 subjects at 6-month follow-up of both treatment groups with gradual relief by the 20-month follow-up. These studies highlighted that although PDT carries the adverse effect of pain, it can potentially be a beneficial adjunct therapy.

### Radiotherapy

Surgical excision of keloids is a potential treatment for mature keloids after failure of first-line therapies. However, as a monotherapy, it is associated with a recurrence rate of up to 100% [87]. To reduce the risk of recurrence, combination treatment modalities have been used. Surgical excision followed by radiation therapy has been showed to be highly effective at reducing recurrence (Table 6). Reduction in fibroblast proliferation and suppression of collagen synthesis by downregulation of TGF-beta and histamine release from mast cells is thought to be the underlying mechanism of action. Typical side effects include dyschromia and telangiectasia.

**Table 6:** Radiotherapy

First author, year	Study design	Treatment	Duration	N	Outcome(s)	Follow-up time	Adverse events	Comments
Aluko-Olokun, 2014 [88]	RCT	IL TAC 10 mg/cm vs excision with 16 gray (Gy) RT (electron)	Q2 weeks for 6 months vs 4 Gy daily for 4 days starting immediately post-op	107	Flattening in 81% vs 58% remained flat (*p* < 0.01)	18 months	Hypopigmentation, ulceration, hyperpigmentation, atrophy, telangiectasia vs pruritus, tenderness, hyperpigmentation	Pinna lesions were 80% of lesions not cured by IL TAC
Dunst, 2013 [89]	P	Excision with brachytherapy of 18 Gy in 3 fractions within first day	All sessions within 36 h of excision	12 (15 keloids)	No recurrence, symptomatic relief	Median 18 months	Hyperpigmentation, hypopigmentation	
Emad, 2010 [90]	P	Excision with 12 Gy radiation (x-ray) vs IL TAC with cryotherapy	4 Gy weekly for 3 weeks starting within 48 hrs vs q20 days until flattening or no response	26 (76 keloids)	Complete remission 70.4% vs 68.8%; partial remission 11.4% vs 3.1%; failure 18.2% vs 28.2%; complete or partial patient satisfaction 100% vs 88.9%	Mean 19 months	25% vs 59.4%: hyperpigmentation, hypopigmentation, telangiectasia, infectious/wound dehiscence vs hypopigmentation, ulceration/necrosis, telangiectasia	
Gupta, 2012 [91]	P	Re-188 skin bandage	2 days	6	1 patient with resolution, 5 patients decrease in size and flattening	3 months	No toxicity	
Gupta, 2013 [92]	RCT	P-32 versus Re-188		16 (42 keloids)	77% vs 59% with > 50% flattening (*p* = 0.664)	Median 6 months	Radiation dermatitis, no difference between groups	
Gupta, 2017 [93]	P	Re-188	3 sessions qod daily	11 (33 keloids)	No recurrence, 67% had > 50% decrease in size	3 years	Radiation dermatitis	
Hafkamp, 2017 [94]	P	Excision with 13 Gy from implanted catheter within 2-h post-op	1 day	24 (29 keloids)	Recurrence rate of 24.1%, POSAS mean of 24.3	> 1 year, median 53 mo	Infection, chronic wound, dehiscence, hyperpigmentation	Only 24 of the 61 patients invited participated
Jiang, 2016 [95]	P	Excision with 18 Gy in 3 fractions within 6-h post-op	3 doses of 6 Gy over 36 h	24 (32 keloids)	Recurrence rate of 6%	Median 29.4 mo	Hypopigmentation, hyperpigmentation, delayed wound healing	
Jiang, 2018 [96]	P	Excision with 18 Gy in 3 fractions within 6-h post-op	3 doses of 6 Gy over 36 h	29 (37 keloids)	Recurrence rate of 8.1%, hypertrophied scars 5.4%	Median 5 years	Delayed wound healing, hyperpigmentation, hypopigmentation, telangiectasia	
Jones, 2019 [97]	P	Excision with up to 18 gy starting 24-h post-op	Up to 18 Gy divided over 4 days	48	19% recurrence	12 months	None reported	
Khalid, 2018 [110]	RCT	Excision with IL TAC/5-FU vs excision with 10 Gy total started within 24-h post-op	Q1 month until resolution vs two consecutive days (5 Gy each)	60	73.33% vs 43.33% had no recurrence after 6 months (*p* = 0.01)	6 months	Skin epidermolysis, wound dehiscence versus skin redness	Keloids on the ears
Kim, 2012 [98]	P	12 to 15 Gy divided into 3 fractions started within 24 h of excision		26	77% complete response	19–36 months	hyperpigmentation	Cesarian section keloids
Lee, 2015 [99]	P	Excision then 12–18 total Gy started within 24 vs 24–72 vs > 72-h post-op	3–4 Gy every other day	30 (37 keloids)	7 recurred, 1 treated within 24 h and 6 treated > 72 h (*p* < 0.0001)	9–51 months	Erythema, hyperpigmentation	
Li, 2014 [100]	RCT	Excision, split thickness graft, 900-cGy radiotherapy vs precut, radiotherapy, excision with split thickness graft, post-RT	RT 10–14 days post-op and repeat 7 days vs RT prior to full excision with repeat 10–14 days post-op	53	55.2% vs 16.7% recurrence; 48.3% vs 8.3% dissatisfied with aesthetic results	12 months	Not stated	Chest wall keloids
Li, 2017 [101]	P	Precut, 900-cGy radiotherapy, excision with graft and repeat 900-cGy radiotherapy if graft survived		86	12.79% recurrence	24 months	Pruritus	Chest wall keloids
Liu, 2018 [102]	p	Keloid scar dissected from keloidal skin used as flap, post-op radiotherapy, hypobaric O2, silicone sheet, and pressure bandage	900-cGy radiotherapy at days 1 and 7 post-op, HBO at day 2 and cont daily until suture removal, silicone and pressure for 6–12 months	45	11.1% recurrence; 84.4% patients satisfied	Mean 18 months	Dyschromia, telangiectasia	Facial keloids
Masoodi, 2014 [103]	P	Excision, 40 mg/mL IL TAC, split thickness graft, one dose 10-Gy radiotherapy within 20-h post-op	12 weeks of silicone sheeting started 3 weeks post-op, plastic clip if VSS > 5 after 12 weeks	24	12.5% recurrence rate, 8.3% with VSS of > 10, mean VSS post-op 4.92 vs pre-op 10.37	>12 months	Hematoma, infection, skin graft loss, regrafting, dyschromia, vascularity, pruritus	Auricular keloids
Mohammadi, 2013 [104]	P	Excision, RT within 24-h post-op	3 Gy daily for 5 days	17 (26 keloids)	VSS pre-op 11.35 vs post-op 3.88 (*p* < 0.005), no recurrence after 16 months	> 11 months	No complications	
Song, 2014 [105]	P	Excision then 10-Gy RT within 72 hrs, pressure, tranilast	Pressure and tranilast for > 3 months	12 (16 keloids)	No recurrence	Mean 20 months	Hyperpigmentation	
Van Leeuwen, 2014 [106]	P	Excision, 12-Gy RT	6-Gy RT within 4 hrs and 6 Gy within 24 hrs	43 (67 keloids)	3.1% recurrence; POSAS physician 16.71 and patient 19.69 mean post-op (range 0 to 60 worst)	Mean 33.6 months	Post-op infection, hypopigmentation, hyperpigmentation,	
Vera, 2019 [107]	P	Excision with brachytherapy catheter 12 Gy in 4 fractions started within 90 min	Q12 hrs	51 (61 keloids)	4.9% recurrence	Median 48 months		Chest
Vila Capel, 2015 [108]	P	Excision, 15 Gy (electron beam) over 5 fractions started within 4-h post-op using aluminum spoiler	5 fractions of 300 cGy over 1 week	19 (20 keloids)	76% no recurrence at end of follow-up	12–68 months	Itching, pain, hyperpigmentation telangiectasia	
Zeng, 2017 [109]	P	Precut, pre-RT w/in 24 hrs, excision with SCIP flat, post-RT at 900 cGy	Pre-RT of 900 cGy twice, second dose post-op day 7	12	Only 1 patient with mild hypertrophic scar	9–24 months	Hyperpigmentation	Presternal keloids

*P* prospective trial, *RCT* randomized controlled trial, *IL* intralesional, *TAC* triamcinolone acetonide, *5-FU*5-fluorouracil, *qn* every n, *RT* radiotherapy, *RF* radiofrequency, *Gy* gray, *cGy* centigray, *hrs* hours, *mo* month

Direct comparisons of methods of keloid treatment are lacking. Aluko-Olokun et al. showed that IL TAC was superior to excision + RT in flattening facial keloids [88]. Similarly, Khalid et al. showed keloids treated with excision followed by IL TAC and 5-FU recurred in 8 of 30 subjects compared to 17 of 30 keloids treated with excision + RT at 6 months [110]. In contrast, Emad et al. found lower treatment failure and higher patient satisfaction with excision + RT than IL TAC and cryotherapy [90].

The majority of studies of excision + RT show administration of radiation within 24 h. Lee et al. compared timing of RT after excision. Of 37 keloids treated, 7 recurred with 1 being treated within 24 h and the other 6 treated after 72 h [99]. There have been a range of radiation doses and schedules investigated in the treatment of keloidal scars with no clear consensus on optimal dose and schedule. Recent evidence examining outcomes of keloids treated with excision and radiation therapy has recurrence rates ranging from no recurrence of the 26 and 16 treated keloids [104, 105] to 56.6% recurrence in 30 treated keloids [110]. Jiang et al. (2015 and 2018) showed low recurrence rates of 2 of 32 treated keloids (6%) and 3 of 37 keloids (8.1% )[95, 96], and Dunst et al. (2013) showed no recurrence with excision followed by 18 Gy of RT in 3 fractions over 36 h [89]. With the same total dose of radiation, Jones et al. showed a recurrence rate of 19% with RT divided over 4 days [97]. In another more extended schedule of radiation, Mohammadi et al. showed no recurrence over a minimum follow-up of 11 months for keloids treated with excision followed by 3 Gy of radiation daily for 5 days [104]. Vila Capel et al. demonstrated a higher 24% recurrence for excision followed by 15 Gy of radiation over 5 fractions given over 1 week using an electron beam with a novel aluminum spoiler [108].

Van Leeuwen et al. found a recurrence rate of 3.1% with excision followed by 12 Gy of RT in two fractions within 24 h [106]. In contrast, 12–15 Gy of radiation divided into three fractions started within 24 h of excision for repeat C-section keloids showed a recurrence rate of 23% (Kim 2012) [98]. A single 13-Gy dose of brachytherapy within 2 h of excision from an implanted catheter also showed a similar rate of recurrence of 24% (Hafkamp 2017) [94]. Vera et al. showed a recurrence rate of 4.9% with excision followed by 12 Gy of brachytherapy in 4 fractions every 12 h (Vera, 2019) [105]. Song et al. also investigated a single radiation dose, showing no recurrence with excision followed by one dose of 10 Gy of radiation within 72 h and continued pressure therapy and oral tranilast (no dose specified, approved in Japan and South Korea) for greater than 3 months [105]. Combination of therapies showed a recurrence rate within the range seen for either excision or RT. Using a combination of excision, intraoperative intralesional triamcinolone, one dose of 10 Gy of radiation within 20 h of excision, and 12 weeks of silicone sheeting with pressure therapy if VSS was > 5 was shown to have a recurrence rate of 12.5% for auricular keloids (Masoodi 2014) [103].

Examining specifically chest wall keloids, studies have focused on precut and pre- and post-RT methods. Zeng et al. showed only one subject with mild hypertrophic scaring after a protocol of precutting for excision, two doses of pre-radiation, excision with flap repair, and post-op RT [109]. Li et al. compared a similar precut method to more conventional excision + radiation for treatment of chest wall keloids [100]. The pre-cut, pre-RT method was superior with a 16.7% recurrence rate compared to 55.2% with only post-excision radiation. In a larger study of this technique, Li et al. demonstrated a recurrence rate of 12.79% over 24 months of follow-up using the precut, pre-radiation method [101].

Liu et al. demonstrated a novel surgical technique of dissecting the keloid tissue from the overlying skin for use as a flap during repair [102]. Excision was followed by RT at days 1 and 7 post-op and hyperbaric oxygen at day 2. Continued silicone and pressure bandaging was used for 6–12 months. Over 18 months of follow-up, the recurrence rate was 11.1%.

Radiation as a monotherapy has also been investigated in the form of personalized patches containing either rhenium-188 or phosphorus-32. Subjects have generally shown flattening of their treated keloids with 59–77% showing > 50% flattening, with the highest percentages in those treated with a P-32 patch [91–93]. The side effects of treatment were radiation dermatitis, which was no different between the P-32 and Re-188 patches.

### Silicone and pressure

Alteration of mechanical forces such as application of pressure or reduction of wound tension has been a long-standing treatment for keloids (Table 7). There has been sparse research examining the use of pressure as a monotherapy for keloids. One such study was a prospective noninvasive intervention study examining the daily application of traditionally worn tight clothing for 2 years conducted by Aluko-Olokun et al. [111] A mean volume reduction of 66.8% was seen in keloids with pedunculated lesions and 100% in keloids in sessile lesions. This study highlights the possible effectiveness of tight clothing as a noninvasive therapy for keloids, especially those with sessile morphology.

**Table 7:** Silicone and pressure dressings or devices

First author, year	Study design	Treatment	Duration	*N*	Outcome(s)	Follow-up time	Adverse events	Comments
Aluko-Olokun, 2017 [111]	P	Daily application of traditionally worn tight clothing	2 years	14 (18 keloids)	Mean 66.8% volume reduction in pedunculated lesions, 100% in sessile	2 years	Not stated	
Bae-Harboe, 2014 [112]	P	IL collagenase then compression earrings	7 h daily for 10 months	6	Average 50% reduction	12 months	Injection site swelling, tenderness, and one ulceration	Earlobe 3 subjects opted for excision at 6, 8, and 11 mo
Bran, 2012 [113]	P	Excision + IL TAC injection then custom-made pressure device	IL TAC Q4–8 wks total 6; pressure device worn o/n 5 nights/wk; adjusted Q4–8 weeks until resolution or after 2 adjustments without improvement	7	No recurrence; all patients satisfied	Mean of 24 months	One patient with Fitzpatrick skin type 5 experienced dyspigmentation from steroid injection which did not resolve	
Carvalhaes, 2015 [114]	P	IL TAC (40 mg/ml, 20 mg/ml or 10 mg/ml) 3 monthly injections prior to excision, one perioperatively, and 2 monthly injections after	8-month total, pressure earrings used 18 h per day for 4 months	46 subjects (81 earlobe keloids)	Injections at 20 mg/ml and 40 mg/ml were effective with no difference between groups (*p* = 0.58)	24 months	Anaphylactic reaction, itching	Patients with 10 mg/ml had poor involution, and this group was stopped
Chen, 2020 [115]	P, SBO	Excision + continuous tension offloading device (TOD)	6 months	38 subjects	3 subjects (7.9%) recurrence 1 unsatisfied	2 years	Mild skin reactions with erythema, pruritus, and tension vesicle	
De Sousa, 2014 [116]	P	Excision with intraoperative and postoperative IL TAC (10 mg/ml) then silicone sheet pressure dressing	Pressure dressing post-op for 48 h then 12 h qnight for 3 months TAC Q3 wks for 12 wks	10 (22 ear keloids)	9.1% recurrence High rate of patient satisfaction compared to physician assessment	16 months	Steroid-induced gastritis, menorrhagia, telangiectasia, and pigmentation	
Hatamipour, 2011 [117]	DB RCT	Excision + topical silicone w/or w/o adjuvant IL 5-FU	Topical silicone 6–12 months Adjuvant 5-FU weeks 1, 2, and 4 and then months 2 and 3	50 subjects	75% of cases w/IL 5-FU were keloid-free, 21% had partial, and 4% no improvement vs 43%, 35%, and 22%	12 months	Pain at injection site, ulceration, burning was not significantly different between the two groups	
Park, 2017 [84]	P	Excision then magnets and silicone gel sheeting pressure therapy	12 hrs/day for 4 months	36 (40 keloids)	Recurrence-free rate of 95.0%	18 months	Not stated	Helical rim keloids
Tanaydin, 2014 [118]	P	Excision then custom molded pressure clip	12–16 hrs/day for 12–15 months	28	71% treated successfully, 29% recurrence	Mean of 8.5 years	61% with discomfort alleviated by adjustment	Nonrecurrence group higher compliance (55% > 12 hrs of wearing) vs recurrence group (38%)

*P* prospective trial, *SBO* single-blinded observer, *DB* double blinded, *RCT* randomized controlled trial, *IL* intralesional, *TAC* triamcinolone acetonide, *qn* every n, *5-FU* 5-fluorouracil, *hrs* hours, *mo* months

Wound tension has been implicated in the pathogenesis of keloid formation. Chen et al. examined the use of a tension offloading device (TOD) applied for 6 months immediately after surgical excision [115]. After 2 years of follow-up, 35 of 38 subjects achieved healing with no recurrence. The use of the TOD requires high patient compliance. According to the authors, the 3 subjects that experienced recurrence in the study were noncompliant with recommended guidelines for TOD use.

A prospective observational study by Tanaydin et al. followed 28 subjects that underwent surgical excision followed by application of a custom molded adjustable pressure clip to be worn 12 to 16 h per day for an average of 12–15 months [118]. In the group that reported nonrecurrence (71%), subjects were more compliant with therapy compared to the recurrence group. Another method of applying adjustable pressure is through magnets as studied by Park et al. where the outcomes of 40 subjects undergoing surgical excision of pure helical rim keloids followed by silicone gel sheets sandwiched between magnets for 12 h a day for 4 months were recorded [82]. At 18-month follow-up, there was a recurrence-free rate of 95% alongside a significant reduction in pain, itch, stiffness, thickness relief, and pliability on POSAS; no adverse events were reported.

The use of adjuvant therapy following surgical excision and application of pressure dressings has also been studied. Hatamipour et al. preformed a double-blinded randomized control trial comparing surgical excision with topical silicone vs adjuvant treatment with 5-FU [117]. At 1-year follow-up, 75% of subjects receiving all three therapies were keloid-free. Similarly, there have been studies examining adjuvant TAC injection with pressure therapy. De Sousa et al. performed a study examining surgical excision with intraoperative and postoperative TAC injection every 3 weeks for 12 weeks as well as silicone pressure dressing applied postoperatively for 48 h [116]. Keloid recurrence of 9.1% was seen at the end of follow-up at 16 months. Carvalhaes et al. also examined the use of intralesional TAC given before excision, perioperatively, and postoperatively [114]. Pressure earrings were used following excision in all groups. IL TAC at 20 mg/ml and 40 mg/ml were effective with no difference between groups. In a study by Bran et al., 7 subjects that underwent surgical excision of auricular keloids with corticosteroid injection followed by application of a custom-made pressure device had complete resolution with no recurrence at 2-years follow-up [113]. Bae-Harboe et al. examined injection of collagenase *Clostridium histolyticum* to earlobe keloids followed by use of compression earrings [112]. An average of 50% reduction was seen in all keloids.

### Other treatments

Recent prospective studies have focused on novel treatment methods (Table 8). Extracorporeal shockwave therapy (ESWT) as a monotherapy for keloids showed a reduction in volume, height, and appearance that was not significantly different compared to intralesional triamcinolone [119]. When ESWT was combined with IL TAC, Kim et al. noted a significant improvement in VSS compared to IL TAC alone, with no significant difference in side effects [120]. Further long-term studies of the effect of ESWT would be interesting as an additional treatment modality prior to excision. Application of a drug-free solid microneedle array found that after 4 weeks of treatment, there was a transient decrease in volume without a difference in VSS compared to an untreated control [121]. The treatment modality was well tolerated, but given that the volume improvement was lost, it is unclear what, if any, therapy duration would be needed for a durable clinical response. Finally, a custom radiotherapy patch led to durable symptomatic improvement and reduction in size in elevation [122]. Further studies will be needed to show how well these patches perform compared to standards of care such as IL TAC. Radiofrequency, most often used in cosmetic procedures such as micro-needling as well as ablative procedures for malignancy, was combined with IL TAC for the treatment of keloids. Weshay et al. treated 21 subjects with 3 to 4 sessions of radiofrequency and then IL TAC, and of the 18 subjects who completed the study, there was a 95.4% reduction in mean volume [123].

**Table 8:** Other treatment modalities

First author, year	Study design	Treatment	Duration	*N*	Outcome(s)	Follow-up time	Adverse events	Comments
Berman, 2013 [124]	P	Excision then porcine gelatin-dextran hydrogel scaffold	NA	19 (26 keloids)	7.7% recurrence rate, average patient scar satisfaction 9.9/10	12 months	None	Earlobe keloids
Bhusari, 2017 [122]	P	Re-188 custom RT patch	3 h on weeks 1 and 3	12 (85 keloids)	Durable symptomatic relief, scar size, and elevation reduced for all	12 months	Ulceration, hypopigmentation	No objective measurements reported
Garakaparthi, 2016 [125]	P	Excision then hydrogel scaffold	NA	19 (26 keloids)	19.2% recurrence rate	12 months		Earlobe keloids
Kim, 2020 [120]	RCT	IL TAC and extracorporeal shockwave therapy (ESWT) vs IL TAC alone	4 sessions of Q3 week IL TAC then ESWT weekly for 10 sessions vs 4 sessions of q3 week IL TAC	40	Mean VSS 7.5 to 3.30 vs 6.85 to 4.1	12 weeks	Telangiectasia, hypopigmentation, atrophy, crystal formation (no difference between groups)	
Limthanakul, 2020 [126]	RCT	Excision then IL TAC (10 mg/ml) versus excision then topical 5% imiquimod cream	IL TAC until scar flattened vs qod for 12 weeks	30	Recurrence 50% versus 21.43%; no significant difference in VSS or patient satisfaction	> 12 months	Itching with imiquimod	Earlobe keloids
Salunke, 2014 [127]	RCT	Ksharsutra ligation vs ksharsutra ligation with agnikarma (cauterization)	NA vs agnikarma on day 3 after keloid removal	20	70% vs 10% recurrence	36 months		Ear pinna keloids, ksharsutra is surgical thread coated with latex of *Euphorbia neriifolia* and *Curcuma longa* powder
Sigler, 2010 [128]	P	2-mg colchicine daily then excision	1 month prior until 1 year after excision	10	No recurrence	2 years	Diarrhea necessitating dose reduction to 1 mg	
Song, 2018 [129]	RCT	Excision, RT, then hyperbaric oxygen therapy (HBOT) vs excision and RT	900-cGy RT on days 1 and 7; HBOT 120 min daily for 2 weeks	240	5.97% vs 14.15% recurrence (*p* < 0.5), 88.81% vs 75.47% fully cured	Median 20.5 vs 21 months	Not stated	
Tan, 2018 [121]	SB, P, intraindividual controlled	Drug-free solid microneedle array (MNA) vs no treatment	4 weeks of treatment	28	Transient decrease in volume, no difference in VSS between treated and untreated	8 weeks	Not stated	1 patient did not participate; volume decreased after treatment but increased 4 weeks after treatment stopped
Wang, 2018 [119]	RCT	ESWT vs IL TAC (10 mg/ml)	3 sessions ESWT in 6 weeks vs IL TAC q2 weeks for 3 sessions	39	Reduction in volume, height, appearance without significant difference between treatments	48 weeks	Not stated	
Weshay, 2015 [123]	p	3–4 sessions of RF then IL TAC (10 mg/mL)	IL TAC q3 months for 3 sessions and once after another 6 months	18	Volume reduction 95.4% (*p* = 0.001); 10% recurrence which resolved with IL TAC	5 years	No infection	

*P* prospective trial, *SB* single blind, *RCT* randomized controlled trial, *IL* intralesional, *TAC* triamcinolone acetonide, *qn* every n, *ESWT* extracorporeal shockwave therapy, *RT* radiotherapy, *RF* radiofrequency, *MNA* microneedle array, *HBOT* hyperbaric oxygen therapy

Many new treatment modalities were investigated as adjunctive therapy with excision. Oral colchicine taken 1 month prior to excision until 1 year after impressively found no recurrence during the follow-up period, though only 10 subjects were treated (Sigler 2010) [128]. Excision with IL-TAC until scar flattening was compared to post-excision 5% topical imiquimod every other night for 12 weeks, showing a reduction in recurrence from 50 to 21.43% [126]. Berman et al. found a very promising recurrence rate of 7.7% for keloids treated with excision and then placement of a porcine hydrogel scaffold [124]. Similarly, Garakaparthi et al. showed a 19.2% recurrence rate with excision and then administration of a hydrogel scaffold for treatment of ear lobe keloids [125]. To improve upon the low recurrence rates of excision followed by RT, Song et al. investigated the addition of hyperbaric oxygen therapy daily for 2 weeks in addition to excision and RT and found it reduced the recurrence rate to 5.9% compared to 14.15% with excision and RT alone [129]. Lastly, Salunke et al. showed that a ligation with cauterization method reduced the recurrence rate from 70% with ligation alone to 10% [127].

## Discussion and recommendations

Pressure and silicone-based therapies have long-standing data behind their efficacy and safety when used both as prevention after surgery and treatment of established keloids, as has been noted by multiple recent consensus guidelines [130]. Recent evidence contributes similar results to the collective literature, showing silicone dressings decreased recurrence while being both safe and well tolerated. Only one recent study examined pressure therapy without excision. Bae-Harboe showed a 50% improvement with pressure earring applied after intralesional collagenase administration. Flatter lesions would likely respond better in combination with corticosteroid impregnated tape and silicone dressings [131]; however, no recent studies have compared these modalities. Overall, these studies highlighted that the key to effectiveness of compression therapy may lie in compliance as well as providing adequate levels of pressure. Limitations to pressure therapy include conspicuous nature of devices, keloid morphology, and patient comfort. Pressure therapy may provide some effect for those looking for conservative treatment for keloids, but effectiveness is increased with combination therapy and with adjustable pressure devices worn for at least periods of 12 h. As ways to manipulate mechanical pressure to treat keloids are explored, the reduction of tension utilizing special tension offloading devices shows promise.

For established keloids, intralesional corticosteroids are the first-line treatment with or without additional therapeutics topically or intralesionally, as is recommended by many consensus guidelines [131–134]. Recent studies have focused on how best to administer IL TAC. Optimal interval timing between injections was suggested to be 2 weeks, though standard of care is typically 4–6 weeks, so further studies confirming this will be needed to change clinical practice. As clinically suspected, sessile lesions were found to respond better to IL TAC compared to pedunculated keloids. The role of IL TAC as an adjuvant to surgical excision continues to have conflicting results in the literature, and some studies lack a control group, making it difficult to recommend compared to methods such as excision with adjunctive RT, which has consistently low recurrence rates.

Other intralesional injections including botulinun toxin A (BTA), bleomycin, mitomycin C, PRP, and collagenase have been recently investigated. The success of treatment with verapamil is mixed and treatment both as an intralesional therapy or as an adjuvant to cryotherapy or excision; verapamil has not consistently outperformed IL TAC. However, verapamil is well-tolerated, so likely lower risk of adverse events. 5-FU has been extensively studied, and recent literature has confirmed synergy in treatment with IL TAC, outperforming either treatment alone in multiple comparative studies, though does have an increased risk of ulceration. 5-FU tattooing has shown promising results, outperforming IL-TAC in a randomized double-blinded study. In recent studies, bleomycin did not outperform IL TAC and had an increased risk of bulla and ulceration. Interestingly, BTA outperformed 5-FU alone and was found to have no difference compared to IL TAC in a double-blinded study, with a low risk of hypopigmentation. Surgical excision with adjuvant cryotherapy and PRP showed a recurrence rate of 16.21%, though since the study had no control group, further study is needed to recommend PRP.

Intralesional cryotherapy is recommended for smaller lesions [131]. Recent comparative studies have shown that intralesional cryotherapy was less effective than excision + IL TAC or excision + RT for resistant keloids, leading to early termination of the trial. Intralesional cryotherapy was shown to have better clinical improvement in two recent studies. Intralesional cryotherapy is a better option for keloids with greater thickness and are not optimal candidates for excision.

Light-based treatment, most commonly PDL or ablative laser therapy, has been recommended as a second-line therapy prior to excision [132]. Fractional CO2 showed no difference in improvement compared to IL verapamil or TAC, and efficacy of CO2 laser with IL TAC compared to cryotherapy with IL TAC was not significantly different. Given the cost and access barriers, laser is likely best in combination with IL or topical CS therapy for the best clinical outcomes shown by multiple recent studies showing improvement with laser treatment followed by IL TAC and/or 5-FU [79–81]. Laser-assisted delivery of corticosteroids and combination of different lasers for treatment of keloids are emerging treatments. Recent studies have shown comparable or slightly improved results with Er:YAG or CO2 followed by topical corticosteroid and occlusion as compared with IL TAC alone or IL TACwith laser. PDT is another emerging application in the field of keloid treatment, though excision followed by PDT has not been found to be more effective than RT.

Excision followed by radiation therapy has been shown to consistently reduce the risk of recurrence. Comparison showed a higher response rate and lower adverse effects compared to cryotherapy with IL TAC. Brachytherapy and externally applied radiation have both shown success with no head-to-head trials. Most successful RT protocols deliver 12–18 Gy over 3–5 days with the optimal timing of radiation beginning within 24 h of excision. For pre-ternal keloids, a specialized method of pre-cut for excision followed by pre-radiation and post-radiation after excision showed a significantly reduced recurrence rate compared to excision with post-radiation only. Radiation therapy alone has shown symptomatic improvement and some success in flattening lesions, but recent studies have not compared it to other first-line therapies such as IL TAC.

Recent investigations of novel treatments have had some promising results. Application of a hydrogel scaffold after excision had low recurrence rates, though have not yet been compared in randomized comparative trials. Both drug-loaded and drug-free microneedle arrays have been tried as a less invasive and painful option, but the clinical improvement has not been shown to be durable as a monotherapy. ESWT with and without IL TAC has been shown to have similar results to IL TAC, which shows promise and warrants further investigation. Topical imiquimod after excision was shown to have reduced recurrence compared to excision with IL TAC, which is a good option for accessible lesions such as ear keloids. Colchicine as an oral therapy started 1 month prior to excision showed no recurrence and was well tolerated, which is a promising systemic therapy option.

### Limitations

Although potential treatments for keloids range from topical and injectable therapeutics to surgical interventions and light therapies, there is no one consistent method of treatment that can guarantee response to therapy and prevent recurrence. Evidence for therapies lack consistent controls, and outcomes are heterogeneous, making it difficult to compare outcomes across studies. Heterogeneity of subject characteristics such as family history, keloid location, skin tension, size, and number, as well as gender and Fitzpatrick skin type, could all play a role in keloid response. There are many novel and effective treatments not included in this review, as non-English language studies, databases from other fields (such as nursing), case studies, case series, and retrospective studies and reviews were excluded from this review of the past decade of investigation. The field of keloid treatment would benefit from consistent, validated outcomes. There are multiple standardized tools for the assessment of keloids including the Patient and Observer Scare Assessment Scale, the Vancouver Scar Scale, and the JSW Scar Scale, and objective measurements of dimensions, color, pliability, and perfusion can be compared [135]. Both subject-controlled and split scar studies are successful controls, and randomization with at least evaluator blinding will improve the quality of evidence. Patient satisfaction and quality of life can also be assessed with the Dermatology Life Quality Index.

## Conclusions

Keloids are a pathologic scarring response to dermal injury that progress to involve normal tissue outside the original injury and have a significant impact on quality of life. With multiple treatment modalities available, first-line therapy is silicone gel or sheeting with corticosteroid injections for more tumoral lesions or tape for flatter keloids. Providers can consider adjuvant intralesional 5-FU, bleomycin, or verapamil depending on patient preference and side effect profile. Laser therapy can be considered in combination with intralesional injection of corticosteroids or topical steroids with occlusion. For keloids that inadequately respond, excision with RT of 16–20 Gy over a maximum of 5 days started within 24 h can be considered. Additional treatment with silicone sheeting and pressure therapy is reasonable with possible oral colchicine to prevent recurrence. As the field continues to progress in the understanding of keloid etiology, the promise of new therapeutic targets and more specialized treatment regimens emerges.

## Supplementary Information

**Additional file 1.** RoB 2 by each study.

**Additional file 2.** ROBINS-I by each study.
