# Supplementary figures and images for: Keloid treatments: an evidence-based systematic review of recent advances
Source: Syst Rev. 2023 Mar 14;12:42. doi: 10.1186/s13643-023-02192-7 (PMC10012475; doi:10.1186/s13643-023-02192-7)

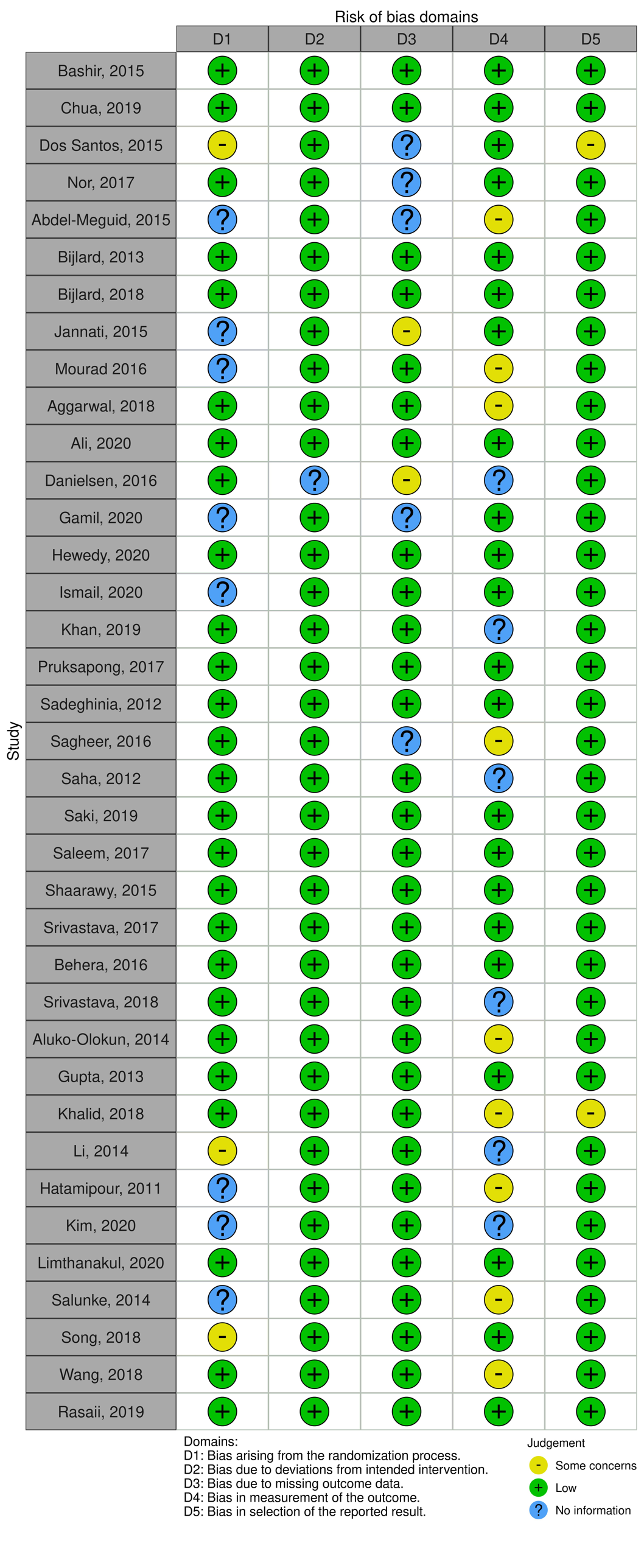

Supplement: Supplementary file 1 — Additional file 1. RoB 2 by each study. [file 13643_2023_2192_MOESM1_ESM.jpg]
